# A short chain fatty acid–centric view of *Clostridioides difficile* pathogenesis

**DOI:** 10.1371/journal.ppat.1009959

**Published:** 2021-10-21

**Authors:** Anna L. Gregory, Daniel A. Pensinger, Andrew J. Hryckowian

**Affiliations:** 1 Department of Medicine, University of Wisconsin School of Medicine & Public Health, Madison, Wisconsin, United States of America; 2 Department of Medical Microbiology and Immunology, University of Wisconsin School of Medicine & Public Health, Madison, Wisconsin, United States of America; 3 Microbiology Doctoral Training Program, University of Wisconsin-Madison, Madison, Wisconsin, United States of America; Geisel School of Medicine at Dartmouth, UNITED STATES

## Abstract

*Clostridioides difficile* is an opportunistic diarrheal pathogen responsible for significant morbidity and mortality worldwide. A disrupted (dysbiotic) gut microbiome, commonly engendered by antibiotic treatment, is the primary risk factor for *C*. *difficile* infection, highlighting that *C*. *difficile*–microbiome interactions are critical for determining the fitness of this pathogen. Here, we review short chain fatty acids (SCFAs): a major class of metabolites present in the gut, their production by the gut microbiome, and their impacts on the biology of the host and of *C*. *difficile*. We use these observations to illustrate a conceptual model whereby *C*. *difficile* senses and responds to SCFAs as a marker of a healthy gut and tunes its virulence accordingly in order to maintain dysbiosis. Future work to learn the molecular mechanisms and genetic circuitry underlying the relationships between *C*. *difficile* and SCFAs will help to identify precision approaches, distinct from antibiotics and fecal transplant, for mitigating disease caused by *C*. *difficile* and will inform similar investigations into other gastrointestinal pathogens.

## Short chain fatty acids are major metabolic end products of gut microbiome community metabolism

The gut microbiome produces a vast array of molecules, which have local and systemic effects on our biology (reviewed in [1,2]). A sum of biogeographical and metabolic factors dictates the nutrients accessible to, and metabolites produced by, this microbial community. While amino acids are less energetically favorable to catabolize than carbohydrates, many gut microbes use amino acids for energy generation via Stickland metabolism [3]. Additionally, fats, proteins, and simple carbohydrates are efficiently absorbed by the host small intestine, leaving few of these metabolites to support the growth of microbes in the large intestine [4,5]. Conversely, complex polysaccharides (e.g., dietary fiber) are not metabolized by the host: The human genome encodes 17 carbohydrate-degrading enzymes, leaving us unable to extract calories from dietary fiber. So, due to our limited repertoire of enzymes, dietary fiber transits to the distal gut and serves as the preferred food source for many members of our microbiome: Gut-resident bacteria of humans and other animals encode tens of thousands of carbohydrate-degrading enzymes capable of breaking various polysaccharide linkages [6].

Monosaccharides that result from dietary fiber degradation by the microbiome enter microbial metabolism via glycolysis or the pentose phosphate pathway and are metabolized to pyruvate. From pyruvate, molecules are further converted into propionate through succinate or lactate, and acetate and butyrate are produced through an acetyl-CoA intermediate or through succinate via the tricarboxylic acid (TCA) cycle [7]. Glutamate and lysine are additional precursors for butyrate, and multiple amino acids are potential precursors for propionate [8]. A substantial amount of cross-feeding occurs among gut microbes. First, primary consumers ferment polysaccharides to lactate, ethanol, or acetate and excrete these metabolites. Subsequently, secondary consumers take up these metabolites and ultimately convert the intermediate products into the short chain fatty acids (SCFAs) propionate and butyrate [9–12]. In general, members of the phylum Bacteroidetes are dominant primary fermenters, which metabolize complex carbohydrates, while the Firmicutes are major secondary consumers [13,14]. In addition, acetate can be produced independently of carbohydrate metabolism by gut microbes via acetogenesis (also known as the Wood–Ljungdahl pathway), which produces approximately one-third of the total acetate in the colon [15].

**Fig 1:**
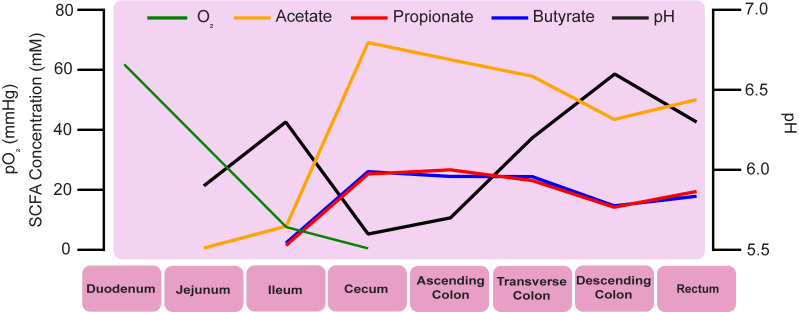
Graphical representation of levels of SCFAs, O_2_, and pH through the longitude of the distal gastrointestinal tract. The green line represents the partial pressure of oxygen (pO_2_ [mm Hg], left axis) through the longitude of the mouse gastrointestinal tract. Levels of oxygen are elevated in the proximal small intestine and are present at negligible amounts in the cecum and more distal portions of the gut. Data on pO_2_ were adapted from [24]. The orange, red, and blue lines represent the concentration of the SCFAs acetate, propionate, and butyrate, respectively, through the longitude of the human gastrointestinal tract (mM, left axis). In general, concentrations of these SCFAs are highest in the cecum and decrease through the more distal portions of the gut. These changes in concentration are a function of absorption by the host and continued production/consumption by gut microbes. The black line represents pH of intestinal contents through the longitude of the human gastrointestinal tract (pH, right axis). Data on SCFA concentration and pH were adapted from [18]. SCFA, short chain fatty acid.

Given the prominence of carbohydrate metabolism by the distal gut microbiome, the SCFAs acetate, propionate, and butyrate are the most abundant microbiome-produced molecules in the gut [12]. Importantly, acetate, propionate, and butyrate comprise >95% of the SCFAs in the gut, while other SCFAs such as formate, valerate, and caproate compose the remaining approximately 5% [16], and the absolute and relative abundance of these SCFAs differ based on host diet and microbiome composition [17]. By extension, low concentrations of SCFAs are often used as a biomarker of a disrupted (dysbiotic) gut microbiome. However, SCFAs are not merely microbial waste products: Substantial amounts are absorbed by host epithelial cells over the length of the colon. For example, one study in humans demonstrated that acetate, propionate, and butyrate are found at concentrations of 69, 25, and 26 mM, respectively, in the human proximal colon and 50, 19, and 17 mM, respectively, in the distal colon ([18] and **Fig 1**). Comparisons of SCFA levels in feces across humans, nonhuman primates, mice, and rats found high levels of SCFAs across all species examined. Therefore, animal models, especially commonly used rodent models, are useful tools to help understand how SCFAs affect microbe–microbe and microbe–host interactions in the human gastrointestinal tract [19].

## Short chain fatty acids have pleiotropic beneficial effects on host biology

SCFAs are rapidly absorbed from the human colon where they can be metabolized by colonocytes or are transported via portal circulation to the liver where they are metabolized or passed into systemic circulation [18,20]. These varied fates of SCFAs underscore the diverse beneficial effects they have on host biology and highlight some of the possible consequences of a microbiome deficient in SCFA production.

Butyrate serves as the primary energy source for colonocytes, providing 60% to 70% of the cell’s energy. In colonocytes, butyrate undergoes β-oxidation to acetate, which enters the TCA cycle [21]. This process requires oxygen, which colonocytes consume from the lumen of the gut, resulting in low concentrations of oxygen in colonic epithelial cells and contributing to the anaerobic environment of the distal gut (**Fig 1** and [22–24]). Low levels of oxygen in the distal gut facilitate the survival of anaerobes, supporting continued gut microbiome community metabolism. Insufficient levels of butyrate shift colonocytes toward anaerobic glycolysis, leading to the accumulation of oxygen in the gut lumen, the loss of epithelial hypoxia, and dysbiosis (reviewed in [25]).

Acetate, propionate, and butyrate are metabolized by host tissues beyond the gut. SCFAs produced by the microbiome enter the liver via the portal vein. In the liver, propionate is utilized as a substrate for gluconeogenesis and acetate as a substrate for de novo lipogenesis and cholesterol synthesis. Propionate and acetate that are not metabolized by the liver enter systemic circulation, where they reach concentrations of up to 1.2 μM and 42 μM, respectively, and are then metabolized by muscle and other tissues [26–29]. The liver also utilizes butyrate for lipid biosynthesis and glycolipid metabolism, though the concentrations of butyrate are low in this tissue, and negligible amounts are found in systemic circulation (reviewed in [25]).

The beneficial impacts of SCFAs extend beyond their roles as metabolites. Acetate, propionate, and butyrate also strengthen the interface between the gastrointestinal lumen and epithelium. Mucus is produced by goblet cells in the gastrointestinal tract, provides a barrier between the lumen of the gut and the colonic epithelium, and is therefore an important component of the mucosal immune system. SCFAs, most notably butyrate, promote mucus production [30,31]. Additionally, SCFAs improve intestinal barrier function by promoting tight junction (TJ) formation [32] and by stabilizing hypoxia-inducible factor (HIF), which is implicated in promoting TJ biogenesis and actin cytoskeletal regulation, to enhance epithelial barrier function [33–35].

Finally, SCFAs act as signaling molecules, which modulate host immune responses through histone deacetylase (HDAC) inhibition and by acting as substrates for G protein–coupled receptors (GPCRs). For example, in peripheral blood mononuclear cells, SCFAs are HDAC inhibitors that modulate immunity by suppressing TNF-α production in an NF-κB–dependent fashion [36,37]. SCFAs are ligands for GPCRs and can influence a range of phenotypes from hormone production to leukocyte development to maintain gut homeostasis [38–40]. There are 3 known intestinal GPCRs that utilize SCFAs as ligands: GPR41 (FFAR3), GPR43 (FFAR2), and GPR109a. Butyrate, propionate, and acetate are agonists for GPR41 and GPR43, while only butyrate activates GPR109a [41,42]. Activation of GPR43 by butyrate is associated with proliferation of anti-inflammatory T_reg_ cells, while SCFA inhibition of HDAC of *Foxp3* is associated with naïve CD4^+^ T cells differentiating into pT_reg_ cells [43–45]. Together, these findings demonstrate that SCFAs, via GPCR engagement, suppress intestinal inflammation to help maintain gut homeostasis

The varied roles of SCFAs in host metabolism, as positive effectors of gut barrier integrity, and as immune regulators support our understanding that the gut microbiome is a key player in maintaining host homeostasis. These observations also indicate the potentially drastic effects that a SCFA-deficient microbiome can have on the host. By extension, the diverse impacts that SCFAs have on host pathways suggest that these metabolites may play comparably important and diverse roles in dictating the biology of gut-resident microbes.

## Short chain fatty acids negatively impact *C*. *difficile* growth

Diverse metabolites in the gastrointestinal tract impact *C*. *difficile* fitness and pathogenesis. For example, bile acids [46,47], metals [48], amino acids [49,50], and sugars [51–53] have a range of effects on *C*. *difficile* in vitro and in animal models of infection. SCFAs are part of the complex metabolic milieu of the gut, and a growing body of literature demonstrates that SCFAs play varied and important roles in the fitness and virulence of phylogenetically diverse bacterial pathogens, including *C*. *difficile*. First, SCFAs have a direct inhibitory effect on the growth of *C*. *difficile*: Acetate, butyrate, propionate, and valerate all reduce the growth rate of *C difficile* in culture [54–56]. Consistent with the in vitro observations that *C*. *difficile* growth is inhibited by SCFAs, in vivo observations illustrate an inverse correlation between SCFAs and *C*. *difficile* pathogenesis [55,56]. The first in vivo connection between SCFAs and *C*. *difficile* pathogenesis was made using a hamster model of infection, where it was observed that older animals (which have elevated levels of SCFAs in their guts relative to younger animals) were less susceptible to *C*. *difficile* infection (CDI) [57]. Indeed, antibiotic exposure, which reduces levels of SCFAs in the gut, is the major risk factor for CDI in humans and is routinely leveraged in animal models to reduce colonization resistance against CDI [51,55,58,59].

Antibiotics are commonly used to treat for individuals with CDI. However, our increasing awareness of the off-target effects that antibiotics have on beneficial microbes has prompted the design and implementation of alternative strategies for CDI. For example, antibiotics such as vancomycin are used to reduce *C*. *difficile* burdens but are often ineffective in resolving recurrent CDI [60], likely because they perpetuate dysbiosis. Furthermore, evidence from murine models of CDI demonstrate that antibiotics can induce a “super-shedder” state in mice, perhaps increasing the transmissibility to new hosts [61], which may be a contributor to the prevalence of CDIs in nosocomial settings. One highly effective alternative is fecal microbiota transplant (FMT), which mitigates recurrent CDI in humans and in animal models, presumably by restoring a “healthy” microbiome [62,63]. FMTs also lead to elevated levels of SCFAs in the recipient, which correlate with reduced *C*. *difficile* burdens and CDI disease symptoms [62]. Another possible alternative to antibiotics for the treatment of CDI is high-fiber dietary intervention, which has shown efficacy in a murine model of CDI [55]. Because dietary fiber impacts the levels of SCFAs and other metabolites in the gastrointestinal tracts of humans and other animals, it represents a promising avenue for restoring healthy microbiome community function and reducing the fitness of *C*. *difficile* in the gut.

### *C*. *difficile* toxins are regulated by metabolic cues, including short chain fatty acids

The pathogenic lifestyle of *C*. *difficile* relies on 2 cytotoxins, TcdA and TcdB (also referred to as “toxins” in this Review), which inactivate Rho GTPases within host colonic epithelial cells, leading the disruption of actin cytoskeletons and epithelial barrier integrity, which ultimately cause epithelial damage and inflammation [64]. Despite the strong association between SCFA levels in the gut and reduced fitness of *C*. *difficile*, previous studies demonstrated that *C*. *difficile* toxin production is elevated in response to SCFAs. Specifically, the addition of acetate, propionate, and butyrate to cultures of *C*. *difficile* stimulates toxin production [55,65]. In addition, metabolic conditions, which lead to elevated intracellular concentrations of butyrate in *C*. *difficile*, are correlated with increased toxin production. For example, intracellular butyrate accumulates and toxins are produced by *C*. *difficile* when cysteine and mixtures of other amino acids are depleted in culture, suggesting that *C*. *difficile* senses and responds to elevated SCFAs and amino acid starvation by increasing inflammation in the gut [65]. Consistent with these observations, high-fiber dietary intervention in mice (which elevates SCFA production by the microbiome) leads to a transient increase in the production of toxins by *C*. *difficile* on a per-cell basis. Importantly, however, the sustained consumption of fiber by the host leads to exclusion of *C*. *difficile* from the community and overall reduction of *C*. *difficile* and its toxins (i.e., an increase in toxin abundance in bulk stool was not observed) [55].

*C*. *difficile* toxin production is controlled by a complex regulatory network. TcdA and TcdB are both encoded in a pathogenicity locus along with several regulatory elements (TcdR and TcdC; [64]). TcdR is a member of the Group V sigma factors and is a positive regulator of toxin expression. TcdR is negatively regulated by TcdC, a dimeric membrane-bound anti-sigma factor. TcdR is responsive to a variety of environmental conditions including temperature, cell redox state, and the abundance of specific intracellular metabolites [66–70].

**Fig 2:**
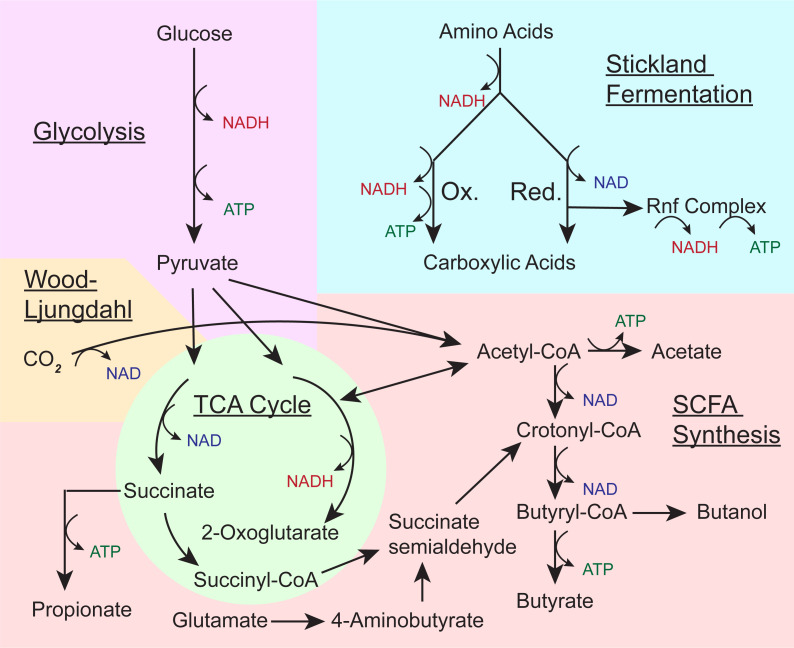
Overview of *C*. *difficile* metabolism with a focus on SCFA synthesis. Glycolysis, Stickland fermentation, TCA cycle, Wood–Ljungdahl pathway, and SCFA synthesis are underlined and presented in a simplified version for clarity; adapted from [77]. Selected precursors, intermediates, and end products of these pathways are labeled with the major precursors and intermediates relevant for SCFA-centric metabolism in *C*. *difficile*. Pathways resulting in net generation of NADH (red), NAD (blue), or ATP (green) are indicated. SCFA, short chain fatty acid; TCA, tricarboxylic acid.

Intracellular metabolites further impact the virulence program of *C*. *difficile* via 4 transcription factors: CodY, Rex, CcpA, and PrdR (reviewed in [71]). These transcription factors sense the abundance of key metabolic inputs like glucose (CcpA) and amino acids (CodY and PrdR) or intracellular redox balance (Rex). Importantly, when each of these regulators is activated, they inhibit *tcdAB* transcription [68,70,72,73]. Although Rex is the only regulator that directly senses NAD/NADH ratios [74], all of these regulators are connected to the maintenance of the NAD/NADH pool via various metabolic steps. For example, one of the major pathways that *C*. *difficile* uses to regenerate NAD is the production of butyrate (**Fig 2**), and both CcpA or CodY directly regulate this metabolism. Specifically, when CcpA or CodY are activated, they inhibit expression of genes involved in the final 2 steps of butyrate production from acetyl-CoA [67,73]. In contrast to butyrate, butanol is an inhibitor of toxin production [65]. CodY, CcpA, and Rex all alter transcription of AdhE, the enzyme responsible for butanol production [67,70,73]. Taken together, these observations link the butyrate/butanol metabolic branch point to the production of toxins by *C*. *difficile*—indicating a positive association between butyrate production by *C*. *difficile* and by the microbiome and *C*. *difficile* pathogenesis.

The conflicting observations that SCFAs impair *C*. *difficile* growth but also increase toxin production create a conundrum on how SCFAs affect *C*. *difficile* pathogenesis. Perhaps exogenous SCFAs impair metabolic functioning of *C*. *difficile*. Given that exogenous SCFAs are a major product of a healthy microbiome, *C*. *difficile* toxin production may be a response to create more inflammation and reduce the fitness of competitors. If so, what are the metabolic pathways that are impacted in *C*. *difficile* in the presence of exogenous SCFAs? Does *C*. *difficile* respond similarly to acetate, propionate, and butyrate or are the responses distinct? By extension, how are these responses linked to toxin production? Finally, why do ecological disturbances that result in large changes in SCFA concentration, such as FMT or high-fiber diets, resolve CDI instead of exacerbate disease? Would interventions that slightly elevate SCFAs have the opposite effect? In order to answer these questions, a more detailed understanding of the links between *C*. *difficile* metabolism and toxin production in the context of complex microbial ecosystems is needed. This will likely be achieved by studies using human samples, animal models of infection, and controlled in vitro systems (e.g., minibioreactor arrays [75], which can be carried out in high throughput while maintaining complex microbial communities).

### *C*. *difficile* has flexible metabolic capabilities, enabling it to occupy various niches

*C*. *difficile* has flexible metabolic capabilities, enabling it to take advantage of different niches during varied dysbiotic states ([59,76]; reviewed in [77] and summarized in **Fig 2**). *C*. *difficile* metabolizes monosaccharides via glycolysis, where it generates pyruvate and acetyl-CoA, both important intermediates in *C*. *difficile* metabolism. Another major energy generating pathway employed by *C*. *difficile* is Stickland fermentation, which is split into oxidative and reductive branches, which share a single initial step, transamination of an amino acid into a 2-oxo-acid [77]. A variety of amino acids are used in the oxidative portion of the pathway, although leucine and isoleucine are preferred substrates. Both NADH and ATP are generated by the oxidative branch of the pathway, and the end product of the pathway is a carboxylic acid with one carbon converted to carbon dioxide [78]. The reductive branch of the pathway regenerates NAD from NADH and produces electrons to be shuttled out of the cell by the RNF complex. Only some specific amino acids (proline, glycine, phenylalanine, tyrosine, and leucine) can be used in the reductive pathway, which similarly results in carboxylic acid end products. *C*. *difficile* has an incomplete TCA cycle that lacks major avenues to produce NADH, and it likely functions to generate metabolic intermediates [79]. Another notable metabolic pathway is the Wood–Ljungdahl pathway in which CO_2_ is reduced to acetate, regenerating NAD in the process [80]. The variety of energy producing pathways available to *C*. *difficile* means that in a dysbiotic gut, *C*. *difficile* can heterogeneously express these different metabolic pathways, enhancing its ability to adapt based on available metabolite pools [59,81].

Like other anaerobes, SCFAs are major metabolic end products of *C*. *difficile* metabolism. Propionate is produced via succinate from the TCA cycle, and acetate is produced either from acetyl-CoA or from the Wood–Ljungdahl pathway. As discussed above, one of the major pathways that *C*. *difficile* uses to regenerate NAD is the production of butyrate. *C*. *difficile* produces butyrate from 3 different precursors: succinate, acetyl-CoA, and glutamate. Butyrate formation via acetate can be coupled to the Wood–Ljungdahl pathway, which provides a highly efficient means for NAD regeneration [80]. Whether butyrate is produced from succinate, acetyl-CoA, or glutamate, these pathways converge on crotonyl-CoA, before a further reduction to butyryl-CoA. Butyryl-CoA is either dehydrogenated to butanol, or more commonly butyrate is produced via 2 alternate reactions with the butyrate kinase reaction also producing ATP [77]. This final step may also be important for replenishing the CoA pool of the cell. Together, the SCFA synthesis pathways, and especially butyrate synthesis, comprise important energy generation and NAD recycling pathways in *C*. *difficile* (**Fig 2**).

## There are several potential mechanisms by which SCFAs may impair *C*. *difficile* growth

*C*. *difficile* is one of many enteric pathogens whose fitness is negatively impacted by microbiome-produced SCFAs (reviewed in [14]). Various mechanisms underlying these effects have been demonstrated for other pathogens, raising many possibilities for how SCFAs impact *C*. *difficile*. For example, SCFAs alter the physical properties of cells. Specifically, in the presence of SCFAs, cytoplasmic pH and osmotic balance are disrupted in *Shigella flexneri*, *Escherichia coli*, and *Salmonella* Typhimurium [82–85]. Excess SCFAs can also interfere with other metabolic pathways or cellular processes, as is the case for acetate-mediated disruption of methionine synthesis or by propionate-mediated inhibition of cell wall and DNA synthesis in *E*. *coli* [86,87]. The observations that different SCFAs impact varied cellular processes within *E*. *coli* are especially important, as they suggest that similarly diverse cellular processes may be impacted by SCFAs in *C*. *difficile*. Furthermore, it is possible that protein activities are directly affected by posttranslational modification (e.g., acetylation, butyrylation, propionylation) [88], which could lead to impaired cellular signaling or enzyme function. Finally, disruption of metabolic processes through product mediated inhibition could be another way in which SCFAs affect *C*. *difficile*, especially for butyrate, as its production can be essential for recycling reducing equivalents in the cell (**Fig 2**). Considering that *C*. *difficile* is sensitive to low pH [89] and that SCFAs are increasingly protonated as pH decreases (and can therefore freely translocate membranes), it is possible that different facets of *C*. *difficile* biology are impacted by SCFAs at different pH values or that pH may amplify the effects of SCFAs (n.b., the pKa for acetate, propionate, and butyrate are 4.76, 4.88, and 4.82, respectively, which are below the typical pH of the human distal gut). Taken together, further investigation into the impacts of SCFAs on *C*. *difficile*, the ways in which these effects impact toxin expression, and the ways in which extracellular pH impacts these responses will enable a better understanding of the varied lifestyles of *C*. *difficile* and ways to mitigate its pathogenesis.

## Toward a better understanding of how *C*. *difficile* balances dysbiosis and inflammation to thrive in the gut

**Fig 3:**
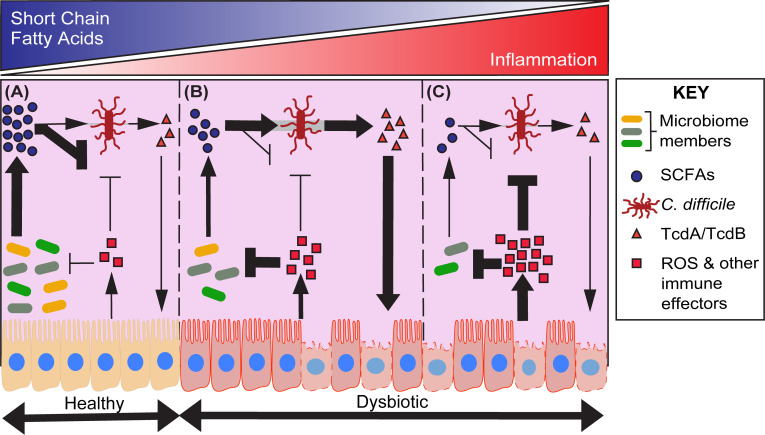
A conceptual model for how *C*. *difficile* balances inflammation and dysbiosis to thrive in the gastrointestinal tract. Key interactions between members of the microbiome, *C*. *difficile*, and the host are included. Pointed arrows indicate positive effects, and blunt arrows indicate negative effects, with their weights signifying their relative importance in each of 3 states: **(A)** A healthy gastrointestinal tract. Gut microbes produce SCFAs as by-products of anaerobic metabolism. These metabolites inhibit *C*. *difficile* growth. In the context of a healthy gut, although SCFAs are also signal for *C*. *difficile* to produce its toxins, SCFA-mediated growth inhibition dominates, and *C*. *difficile* cannot establish a niche. **(B)** A dysbiotic gastrointestinal tract, where *C*. *difficile* thrives. After an ecological disturbance (e.g., antibiotics), *C*. *difficile* is able to proliferate within the gut due to an abundance of available nutrients. However, as the microbiome recovers from the disturbance, the concentration of SCFAs increase, which *C*. *difficile* uses as a signal to up-regulate its toxins. These toxins disrupt host epithelial cells and lead to the production of immune effectors (including ROS), which suppress the recovery of obligate anaerobes (competitors of *C*. *difficile*) in the gut. As a result, despite the presumed negative impacts of ROS on *C*. *difficile*, it maintains its inflammation-associated niche by excluding competing microbiome members. **(C)** A highly inflamed gastrointestinal tract. Here, it is presumed that *C*. *difficile* is unable to tolerate the negative effects of the host immune response and its growth is inhibited. We posit that the efficacy of FMT (in humans and animal models) and dietary intervention (in animal models) is due in large part to these interventions shifting the gut ecosystem from the state illustrated in panel B to the state illustrated in panel A. A better understanding of the transitions between all 3 states represented in the figure (e.g., A↔B, B↔C) will enable precision approaches for mitigating CDI in at risk human populations. CDI, *C*. *difficile* infection; FMT, fecal microbiota transplant; ROS, reactive oxygen species; SCFA, short chain fatty acid.

The combined observations that (1) SCFAs are major metabolic end products of anaerobic metabolism in the gastrointestinal tract; (2) SCFAs play important roles in maintaining gut homeostasis; and (3) SCFAs impact *C*. *difficile* growth and virulence together highlight a conceptual model to guide future work (**Fig 3**).

A healthy gut is a highly competitive environment that resists CDI. In addition to a lack of available nutrients for *C*. *difficile*, high levels of SCFAs likely contribute to the inability of *C*. *difficile* to colonize or to grow to sufficient densities to impact the host (**Fig 3A**). However, after an ecological disturbance, such as antibiotic treatment, *C*. *difficile* populations expand in the gut. Data from humans and murine models of infection demonstrate that, though *C*. *difficile* remains a minority member of the microbiome throughout infection, there is little competition for metabolites (e.g., amino acids, organic acids, and sugars) during this expansion and that *C*. *difficile* produces no detectable toxin [49,53,59,90–92]. As the microbiome recovers, the concentrations of available nutrients decrease, and the concentrations of SCFAs increase. *C*. *difficile* senses and responds to the environmental change by up-regulating its toxins, which elevate inflammation [90]. We hypothesize that SCFAs are a key signal for *C*. *difficile* in this transition and that the resulting toxin-mediated inflammation helps to reestablish facets of community composition and function where *C*. *difficile* thrives (**Fig 3B**).

Importantly, this view of *C*. *difficile* pathogenesis is informed by recent studies focused on pathogenic Enterobacteriaceae (e.g., *Salmonella* Typhimurium, *E*. *coli*), where pathogen-mediated inflammation reduces the fitness of competing members of the microbiome and enables these pathogens to access privileged nutrients and electron acceptors [93–95]. Like the Enterobacteriaceae, *C*. *difficile-*induced inflammation liberates nutrients from the host that *C*. *difficile* can utilize for growth. For example, in a toxin-dependent fashion, *C*. *difficile* uses host-derived collagen breakdown products (which are rich in Stickland substrates like proline, hydroxyproline, alanine, and glycine) and sorbitol (which is used as a carbon source) [52,96]. However, unlike the Enterobacteriaceae, *C*. *difficile* is an obligate anaerobe (i.e., it cannot use O_2_ as a terminal electron acceptor) and is only capable of growing in the presence of relatively low levels of oxygen (<3% O_2_), likely due to a suite of enzymes involved in detoxifying reactive oxygen species [97–99]. So, we hypothesize that if inflammation (*C*. *difficile*-mediated or otherwise) elevates both molecular O_2_ and reactive oxygen species in the gut to high levels, *C*. *difficile* fitness would be reduced (**Fig 3C**).

Taken together, we hypothesize that there is an optimal level of inflammation that allows *C*. *difficile* to outcompete members of the gut microbiome, while being able to survive as an anaerobe in an inflamed environment. This optimal level may vary between hosts, depending on variables like host genetics, lifestyle choices, or microbiome composition. Interindividual variation in this optimal level may help to explain variability in humans who can experience a range of *C*. *difficile*-related colonization states from asymptomatic carriage to recurrent and fulminant colitis. By learning the specific molecular and genetic mechanisms underlying the response of *C*. *difficile* to SCFAs, as well as host- and microbiome-specific factors that influence this response, we expect to be able to develop new concepts and approaches for coping with this pathogen. For example, though dietary fiber intervention has shown promise in reducing burdens of *C*. *difficile* in a mouse model of CDI, the path toward translating these findings to humans is unclear. Are there fiber types that clear CDI in humans while others do not? Are the effective fiber types generalizable to all humans or do dietary interventions need to be personalized based on host genetics or microbiome composition? Could a mix of fiber types or synbiotics (fiber coadministered with specific microbes that consume it) increase generalizability of the intervention? Will patients, especially those who consume low-fiber “Western” diets be able to tolerate fiber treatments or will they require an acclimation period?

Alternatively, it is possible that dietary intervention and other SCFA-focused investigations may simply be a tool to toggle the metabolic landscape of the gut and the fitness of *C*. *difficile* in the laboratory. In this case, by using these powerful tools, along with systems biology approaches, bacterial genetics, and gnotobiotic animal models, a foundation will be built for uncovering specific *C*. *difficile* effectors, microbe–microbe, or microbe–host interactions that can be drugged with novel compounds, next-generation probiotics, or other targeted approaches. Regardless, continued focus on the ways in which *C*. *difficile* responds to SCFAs will yield promising conceptual and practical advances on how we understand and treat this vexing bacterial pathogen.
